# Comparative Study Between Various Scoring Systems in Predicting the Severity of Acute Pancreatitis

**DOI:** 10.7759/cureus.84004

**Published:** 2025-05-13

**Authors:** Shrinath G Deshpande, Manjusha M Litake

**Affiliations:** 1 Surgical Gastroenterology, Bangalore Medical College and Research Institute, Bengaluru, IND; 2 General Surgery, Government Medical College & Hospital, Baramati, IND

**Keywords:** apache ii, bisap, modified ct severity index, necrosis, pancreatitis, ranson

## Abstract

Background

Acute pancreatitis (AP) is an acute inflammatory condition of the pancreas, peri-pancreatic tissues, and several organs, leading to multiple organ dysfunction syndrome and a higher risk of mortality. For many years, scoring systems that include biochemical, radiological, and clinical criteria for determining severity have been used. Though numerous studies have used various scoring methods to evaluate the severity of AP, this study has been conducted to compare four scoring systems: bedside index of severity in AP (BISAP), acute physiology and chronic health evaluation (APACHE II), Ranson’s, and modified CT severity index (CTSI) based on clinical, biochemical, and radiological parameters.

Materials and methods

It was a prospective-comparative study. The study was conducted from December 2016 to August 2018 in the Department of General Surgery at Byramjee Jeejeebhoy Government Medical College (B.J.G.M.C.) and Sassoon Hospital, Pune, Maharashtra, India. A total of 75 participants were enrolled in the study.

Results

The study population ranged from 18 to 68 years, with a mean age of 40.8±11.5 years. AP was most prevalent in the age group of 31-40 years (33.3% cases). Out of 75 patients in this study, 14 patients (18.7%) had severe AP (SAP), 18 patients (24%) had moderate SAP, and 43 patients (57.3%) had mild AP. Ten patients expired, with a mortality rate of 13.3%. It has been observed that the BISAP score had the best specificity (100%) and the CTSI score had the highest sensitivity (96.9%) among our study's four scores for predicting pancreatic necrosis. When predicting persistent organ failure, BISAP had the highest specificity, and Ranson and CTSI scored the highest sensitivity. The modified CTSI poorly predicted AP, patients' mortality, and SAP.

Conclusion

The BISAP score provides a straightforward and accurate way to analyze the seriousness of AP. Ranson’s score is also a reliable indicator of ongoing organ failure among AP cases. The most reliable technique for predicting pancreatitis mortality is the APACHE II score.

## Introduction

Acute pancreatitis (AP) is an acute inflammatory condition of the pancreas, peri-pancreatic tissues, and several organs, leading to multiple organ dysfunction syndrome (MODS) and a higher risk of mortality [1]. The incidence of AP in India has been observed to be 2.6-3.2 cases per 100,000 population [2].

According to the revised Atlanta classification, the three categories that classify AP are mild pancreatitis, moderate pancreatitis, and severe AP (SAP) [3]. Around 80% of patients have a mild attack of pancreatitis, the mortality from which was found to be around 1%, with spontaneous recovery within 3-5 days. AP with systemic and/or local consequences is sometimes called SAP. The term "early severe AP" refers to organ dysfunction that appears within 72 hours of the diagnosis. The main factors that determine death in AP include infection, pancreatic necrosis, early hypoxemia, short course, and increasing MODS [4,5]. SAP patients may die from acute respiratory, cardiovascular, and renal problems. Mortality rates from SAP have been reported to range from 20% to 60%. Sepsis is the main cause of delayed mortality [6].

Premature pancreatic enzyme activation causes local inflammation and autodigestion, which is the basic pathophysiology of AP [7]. Systemic inflammatory response syndrome is caused by these enzymes entering the venous flow and helping in the synthesis of inflammatory cytokines [8].

To determine patients at risk of morbidity and death, it is essential to assess the severity precisely at hospital admission. Many prognostic indicators can be used to classify the severity of AP. There are 11 criteria in the Ranson score [9], 8 criteria in the Glasgow score [10], 5 criteria in the bedside index of severity in AP (BISAP) score [11], 14 criteria in the acute physiology and chronic health evaluation (APACHE II) score [12], and radiological scoring involving modified CT severity index (CTSI) parameters [13]. Based on the cut-off number and scoring timing, for SAP prediction, the sensitivity and specificity of several scoring methods varied from 55% to 90% [14]. These scoring methods have drawbacks; one of them is receiving the outcome 48 hours after admission. It has been shown to have an 87% sensitivity and a 90% overall detection rate for pancreatic necrosis [15]. One of the most useful biochemical tests with a sensitivity of 85% was CRP [16].

The study was conducted to compare four different scoring systems such as BISAP, CTSI, Ranson, and APACHE II scoring to assess the severity of AP.

## Materials and methods

Study design

It was a prospective-comparative study. The study was conducted from December 2016 to August 2018 in the Department of General Surgery at Byramjee Jeejeebhoy Government Medical College (B.J.G.M.C.) and Sassoon Hospital, Pune, Maharashtra, India.

Study population

A total of 75 participants were part of this study. All patients with the mentioned inclusion criteria were admitted to the Department of General Surgery at B.J.G.M.C. and Sassoon Hospital, Pune. Participants who had a clinical history of abdominal pain, elevated pancreatic enzyme levels (serum amylase/lipase >3 times the institution's upper limit), abdominal pain that began within 72 hours; who were above 18 years; and had evidence of an enlarged or edematous pancreas on USG or CT abdomen were all eligible. The exclusion criteria involved participants under 18 years of age, pregnant women, and patients with proven cases of chronic pancreatitis.

Study procedure

After a diagnosis was made, each patient's disease severity was evaluated using the Ranson [9], BISAP [11], APACHE II [12], and modified CTSI [16]. Grading systems and the results were compared using common statistical tools. If there are no indications of alternative etiologies, if the patient regularly consumes large amounts of alcohol each day, or if there was an alcohol binge before the onset of sickness, alcoholic pancreatitis was investigated. According to the history and preliminary studies, idiopathic pancreatitis had no discernible etiological cause. Prospective observations were made on patients throughout their hospital stay until they were discharged or passed away.

Data collection

BISAP, Ranson, APACHE II, and modified CTSI scores were determined. Ranson and APACHE II scores were calculated at admission and within 48 hours. Further, the severity of pancreatitis was graded into mild, moderate, and severe AP. Then, the organ failure was assessed using the Marshal scoring system.

Statistical analysis

The data were analyzed using SPSS, Version 21.0 (IBM Corp., Armonk, NY). Categorical variables were presented as n (%), while the continuous variables were expressed as mean and SD. A comparison among categorical variables was made using the Chi-square test. Cohen's kappa test was used to examine the statistical agreement between the two diagnostic approaches. p-values less than 0.05 were considered to be statistically significant.

Ethical clearance

Ethical clearance was granted by the institutional ethics committee (IEC) of B.J.G.M.C. and Sassoon Hospital, Pune, Maharashtra, India, under letter reference number BJGMC/IEC/Pharmac/D-0217025-025.

## Results

The study population (n=75) ranged from 18 to 68 years, with the mean age being 40.8±11.5 years. AP was most prevalent between 31 and 40 years (33.3% of cases). It was found that of the 75 cases, 43 (57.3%) had mild pancreatitis, 18 (24.0%) had moderate pancreatitis, and 14 (18.7%) had severe pancreatitis, according to the revised Atlanta classification. Among 75 patients, 65 (86.7%) survived and 10 (13.3%) expired. Table 1 represents the characteristics of the enrolled participants.

**Table 1:** Patient demographics Data were presented as either mean±SD or n (%). SD, standard deviation

Characteristics	Values
Age (in years)	40.8±11.5
Male participants	65 (86.7%)
Female participants	10 (13.3%)
Etiology	
Alcoholic	66 (88%)
Gall stones	07 (9.3%)
Idiopathic	01 (1.3%)
Trauma	01 (1.3%)
Severity of pancreatitis (revised Atlanta classification)	
Mild	43 (57.3%)
Moderate	18 (24%)
Severe	14 (18.7%)
Mortality	
Survived	65 (86.7%)
Expired	10 (13.3%)

Of the 75 cases studied, 59 (78.7%) had a modified CTSI score of >4, 25 (23.3%) had a Ranson score of ≥3, 15 (20%) had a BISAP score of ≥3, and 23 (30.7%) had an APACHE II score of ≥8. Table 2 shows the distribution of cases according to cut-off values in various scoring systems.

**Table 2:** Distribution of cases according to cut-off values in various scoring systems Data were presented as n (%). APACHE II, acute physiology and chronic health evaluation; BISAP, bedside index of severity in acute pancreatitis; CTSI, CT severity index

Scoring system	Category	Number of cases
Modified CTSI score	≤4	16 (21.3%)
	>4	59 (78.7%)
Ranson score	<3	50 (66.7%)
	≥3	25 (33.3%)
BISAP score	<3	60 (80%)
	≥3	15 (20%)
APACHE II score	<8	52 (69.3%)
	≥8	23 (30.7%)

Table 3 shows the distribution of the prediction of pancreatic necrosis by various scoring systems used in the study groups. All the scores, including CTSI, Ranson, BISAP, and APACHE II scores, were statistically significant with the prediction of necrosis.

**Table 3:** Distribution of the presence of pancreatic necrosis by various scoring systems used in the study group Data were presented as n (%). The p-value was obtained using the chi-square test. The statistical agreement between the two systems was assessed using Cohen's kappa. p-value of <0.05 was considered significant. APACHE II, acute physiology and chronic health evaluation; BISAP, bedside index of severity in acute pancreatitis; CTSI, CT severity index

Scoring system	Category	Present (n=33)	Absent (n=42)	Cohen's kappa (p-value)
Modified CTSI score	>4	32 (54.2%)	27 (45.8%)	0.3 (0.001)
	≤4	01 (6.3%)	15 (93.8%)	
Ranson score	≥3	19 (76%)	06 (24%)	0.4 (0.001)
	<3	14 (28%)	36 (72%)	
BISAP score	≥3	12 (80%)	03 (20%)	0.31 (0.001)
	<3	21 (35%)	39 (65%)	
APACHE II score	≥8	15 (65.2%)	08 (34.8%)	0.27 (0.01)
	<8	18 (34.6%)	34 (65.4%)	

Table 4 shows the distribution of the prediction of persistent organ failure by various scoring systems used in the study groups. Ranson, BISAP, and APACHE II scores were found to be highly significantly associated with persistent organ failure. Modified CTSI had a low positive predictive value (20.3%) and accuracy (37%) for predicting persistent organ failure. For predicting persistent organ failure, the APACHE II score had the highest accuracy (85.3%), the BISAP score had the highest specificity, and the Ranson score had the highest sensitivity and negative predictive value.

**Table 4:** Distribution of prediction of persistent organ failure by various scoring systems used in the study group Data were presented as n (%). The p-value was obtained using the chi-square test. The statistical agreement between the two systems was assessed using Cohen's kappa. p-value was considered significant at <0.05. APACHE II, acute physiology and chronic health evaluation; BISAP, bedside index of severity in acute pancreatitis; CTSI, CT severity index

Scoring system	Category	Present (n=12)	Absent (n=63)	Cohen's kappa (p-value)
Modified CTSI score	>4	12 (20.3%)	47 (79.7%)	0.09 (0.04)
	≤4	00 (0%)	16 (100%)	
Ranson score	≥3	12 (48%)	13 (52%)	0.55 (0.001)
	<3	00 (0%)	50 (100%)	
BISAP score	≥3	08 (53.3%)	07 (46.7%)	0.5 (0.001)
	<3	04 (6.7%)	56 (93.3%)	
APACHE II score	≥8	11 (47.8%)	12 (52.2%)	0.53 (0.001)
	<8	01 (1.9%)	51 (98.1%)	

There was a statistically significant agreement between the modified CTSI, Ranson, BISAP, and APACHE II scoring systems and the incidence of moderate/severe pancreatitis. Table 5 depicts the distribution of the prediction of the severity of pancreatitis by various scoring systems in the study groups.

**Table 5:** Distribution of prediction of moderate/severe pancreatitis by various scoring systems in the study group Data were presented as n (%). The p-value was obtained using the chi-square test. The statistical agreement between the two systems was assessed using Cohen's kappa. p-value was considered significant at <0.05. APACHE II, acute physiology and chronic health evaluation; BISAP, bedside index of severity in acute pancreatitis; CTSI, CT severity index

Scoring system	Category	Moderate/severe (n=32)	Mild (n=43)	Cohen's kappa (p-value)
Modified CTSI score	>4	31 (52.5%)	28 (47.5%)	0.28 (0.001)
	≤4	01 (6.3%)	15 (93.8%)	
Ranson score	≥3	21 (84%)	04 (16%)	0.57 (0.001)
	<3	11 (22%)	39 (78%)	
BISAP score	≥3	15 (100%)	00 (0%)	0.5 (0.001)
	<3	17 (28.3%)	43 (71.7%)	
APACHE II score	≥8	19 (82.6%)	04 (17.4%)	0.51 (0.001)
	<8	13 (25%)	39 (75%)	

The BISAP score had the highest specificity, whereas the Ranson score was most accurate in predicting moderate SAP or SAP. Table 6 depicts the diagnostic efficacy indices of several scoring systems for the assessment of moderate/severe pancreatitis.

**Table 6:** Distribution of different scoring systems' diagnostic effectiveness indices for predicting moderate-to-severe pancreatitis in the research group APACHE II, acute physiology and chronic health evaluation; BISAP, bedside index of severity in acute pancreatitis; CTSI, CT severity index; NPV, negative predictive value; PPV, positive predictive value

Diagnostic efficacy indices (%)					
Scoring systems	Sensitivity	Specificity	PPV	NPV	Accuracy
Modified CTSI score	96.9	34.9	52.5	93.7	61.3
Ranson score	65.6	90.7	84	78	80
BISAP score	46.9	100	100	71.7	77.3
APACHE II score	59.4	90.7	82.6	75	73

Table 7 shows the distribution of SAP by various scoring systems used in the study group, including the modified CTSI, Ranson, BISAP, and APACHE II scores.

**Table 7:** Distribution of SAP by various scoring systems used in the study group Data were presented as n (%). APACHE II, acute physiology and chronic health evaluation; BISAP, bedside index of severity in acute pancreatitis; CTSI, CT severity index; SAP, severe acute pancreatitis

Scoring system	Category	Number of SAP cases
Modified CTSI score	≤4	00 (0%)
	>4	14 (100%)
Ranson score	<3	01 (7.7%)
	≥3	13 (92.7%)
BISAP score	<3	04 (28.6%)
	≥3	10 (71.4%)
APACHE II score	<8	01 (7.7%)
	≥8	13 (92.7%)

Modified CTSI had the highest sensitivity but poor accuracy (40%) in predicting SAP. BISAP score had the highest specificity and accuracy in predicting SAP. Table 8 depicts the distribution of the diagnostic efficacy of various scoring systems in predicting the severity of SAP.

**Table 8:** Diagnostic efficacy indices of different scoring systems for the study group's SAP prediction APACHE II, acute physiology and chronic health evaluation; BISAP, bedside index of severity in acute pancreatitis; CTSI, CT severity index; NPV, negative predictive value; PPV, positive predictive value; SAP, severe acute pancreatitis

Diagnostic efficacy indices (%)					
Scoring systems	Sensitivity	Specificity	PPV	NPV	Accuracy
Modified CTSI	100.0	26.2	23.7	100.0	40.0%
Ranson score	92.9	80.3	52.0	98.0	82.7%
BISAP score	71.4	91.8	66.7	93.3	88.0%
APACHE II score	92.9	83.6	56.5	98.1	85.3%

The distribution of incidence of mortality was not significantly associated with CTSI scoring, with a p-value of 0.07, while it was highly significantly associated with Ranson, BISAP, and APACHE II scores, with a p-value of 0.001. Table 9 shows the distribution of the prediction of mortality by various scoring systems.

**Table 9:** Distribution of prediction of mortality by various scoring systems Data were presented as n (%). The chi-square test obtained a p-value. The statistical agreement between the two systems was assessed using Cohen's kappa. The p-value was considered significant at <0.05. APACHE II, acute physiology and chronic health evaluation; BISAP, bedside index of severity in acute pancreatitis; CTSI, CT severity index

Scoring system	Category	Expired (n=10)	Survived (n=65)	Cohen's kappa (p-value)
Modified CTSI	>4	10 (16.9%)	49 (83.1%)	0.08 (0.07)
	≤4	00 (0%)	16 (100%)	
Ranson score	≥3	09 (36%)	16 (64%)	0.4 (0.001)
	<3	01 (2%)	49 (98%)	
BISAP score	≥3	06 (40%)	09 (60%)	0.38 (0.001)
	<3	04 (6.7%)	56 (93.3%)	
APACHE II score	≥8	10 (43.5%)	13 (56.5%)	0.51 (0.001)
	<8	00 (0%)	52 (100%)	

## Discussion

The study compared four different scoring systems, including BISAP, CTSI, Ranson, and APACHE II scoring, to assess severity, complications, and mortality due to AP. Among all four scoring systems, Ranson and APACHE II scoring systems were based on biochemical parameters, CTSI was based on radiological parameters, and BISAP was considered a bedside index to estimate the severity of AP.

In our study, AP was most prevalent in the age group of 31 to 40 years. The average age for the study was 40.8 years, which matched the study by Khanna et al. [17]. The incidence of AP was higher in males than in females in the study. A similar study by Singh et al. showed that AP was more prevalent with a ratio of 6:1 in males and females, respectively [18]. In our study, abdominal pain (100%), associated back pain (90.7%), and vomiting (69.3%) were the predominant presenting complaints. Our results were similar to the study performed by Khanna et al. in which abdominal pain was present in 100% of cases and vomiting in 70.8% of cases [17].

Alcohol accounted for 88% of the etiological factors in our study, with gallstones coming in second place. This correlates with the results of Yadav et al. (40.3%) and Simoes et al. (39.3%), in which alcohol was the most common etiology [19,20].

Based on earlier research in this area performed by Khanna et al. and Kumar et al., the disease severity was evaluated using a cut-off of BISAP score ≥3, APACHE score ≥8, Ranson score ≥3, and modified CTSI score >4 [17,21].

In our study, 12 patients (16%) developed persistent organ failure according to the Ranson scoring, while APACHE II and BISAP scoring showed a significant statistical association. Ranson and modified CTSI scores had the highest sensitivity, and BISAP had the highest specificity in predicting persistent organ failure. In a similar study performed by Khanna et al [17], the BISAP score showed a 66% specificity, whereas the Ranson score had the highest sensitivity of 92% and specificity of 74.5%. With a kappa value of 0.552, our study's Ranson score was found to have the strongest connection in predicting persistent organ failure. Research by Khanna et al. stated that the Ranson score had the highest kappa value of 0.61 [17]. BISAP scores showed the highest specificity, and Ranson and CTSI scores had the highest sensitivity in our study. When it came to predicting persistent organ failure, the Ranson score showed the strongest association.

All scores had a significant correlation in predicting SAP in persistent organ failure. The BISAP score was shown to have the highest specificity (91.8%) and accuracy (88%) in predicting SAP in our study, while the modified CTSI (100%), Ranson (92.9%), and APACHE II scores (92.9%) had high sensitivities. Modified CTSI had the lowest accuracy in predicting SAP [17].

Around 10 participants expired, with a mortality rate of 13.3%. Similar mortality rates were seen in studies performed by Yadav et al. [19] at 10.1% and Vasudevan et al. [22] at 18%. Of the 10 patients who died, nine had SAP, and one died of myocardial infarction. One patient of SAP died of MODS on postoperative day 2 following an open necrosectomy. The 10 patients who died in our study had APACHE II scores more than or equal to 8 and CTSI scores more than 4. Ranson scores more than or equal to 3 were found in nine patients, and BISAP was more than or equal to 3 in six patients. In our study, APACHE II, Ranson, and BISAP scores had a strong and significant association in predicting mortality (p<0.001), with APACHE II having the highest accuracy of 86.7% and a sensitivity of 90%. The modified CTSI had a sensitivity of 100%. This is also comparable with Khanna et al. 's study that showed APACHE II score having the highest sensitivity and accuracy in predicting mortality in AP [17].

Limitations

The limitations of this study were that it was single-centric and the study duration was shorter. To generalize the findings, a bigger sample size and a multicentric investigation are necessary. Also, the longevity of the study duration might help in the enrollment of a large number of participants.

## Conclusions

The study showed that the BISAP score was a simple and reliable method to predict SAP, as per the results. Ranson's score was a predictor of persistent organ failure in cases of AP. The APACHE II score was a reliable way to forecast pancreatitis mortality. The BISAP score has the advantage of easy and quick risk stratification, with similar efficacy to complex scores such as Ranson and APACHE II. Modified CTSI had the highest sensitivity in pancreatic necrosis but was not accurate in predicting severity and mortality compared to other scoring systems. However, for further studies, depending on the factors being taken into account, the availability of resources, and local practices, the most helpful scoring tool might be chosen.
